# Supplementary material for: Multication perovskite 2D/3D interfaces form via progressive dimensional reduction
Source: Nat Commun. 2021 Jun 9;12:3472. doi: 10.1038/s41467-021-23616-9 (PMC8190276; doi:10.1038/s41467-021-23616-9)
Supplement: Supplementary file 1 — Supplementary Information [file 41467_2021_23616_MOESM1_ESM.pdf]

Multication Perovskite 2D/3D Interfaces Form via Progressive Dimensional Reduction

Andrew H. Proppe,[1,2,†] *Andrew Johnston,*[2,†] *Sam Teale,*[2,†] *Arup Mahata,*[3,4] *Rafael Quintero-Bermudez,*[2] *Eui Hyuk Jung,*[2] *Luke Grater,*[2] *Teng Cui,*[5] *Tobin Filleter,*[5] *Chang-Yong Kim,*[6] *Shana O. Kelley,*[1,7] *Filippo De Angelis,*[3,4,8,9] *Edward H. Sargent*[2,*]

[1] Department of Chemistry, University of Toronto, 80 St. George Street, Toronto, Ontario, Canada, M5S 3G4.

[2] The Edward S. Rogers Department of Electrical and Computer Engineering, University of Toronto, 10 King's College Road, Toronto, Ontario, Canada, M5S 3G4.

[3] D3-Computation, Istituto Italiano di Tecnologia, Genova, Italy.

[4] Computational Laboratory for Hybrid/Organic Photovoltaics (CLHYO), Istituto CNR di Scienze e Tecnologie Chimiche (CNR-SCITEC), Istituto CNR di Scienze e Tecnologie Molecolari (ISTM-CNR), Via Elce di Sotto 8, 06123 Perugia, Italy.

[5] Department of Mechanical and Industrial Engineering, 5 King's College Road, Toronto, ON M5S 3G8, Canada

[6] Canadian Light Source, 44 Innovation Blvd, Saskatoon, Saskatchewan, S7N 2V3

[7] Department of Pharmaceutical Sciences, Leslie Dan Faculty of Pharmacy, University of Toronto, Toronto, Ontario, Canada, M5S 3M2

[8] Department of Chemistry, Biology and Biotechnology, University of Perugia, Via Elce di Sotto 8, 06123, Perugia, Italy.

[9] Chemistry Department, College of Science, King Saud University, Riyadh, Saudi Arabia

[†] These authors contributed equally to this work.

Contains:

Supplementary Note 1. Materials

Supplementary Figures 1 to 11

Supplementary Tables 1 to 3

Supplementary Note 1

Materials. All chemicals used are commercially available from Sigma-Aldrich (or otherwise specified) and were used without any additional purification steps: 4-vinylbenzylamine (stabilized with MEHQ, >92.0%, TCI (Product Number : V0101)), dimethylformamide anhydrous (DMF, 99%), dimethylsulfoxide anhydrous (DMSO, 99.9%), lead (II) iodide (from Alfa Aesar, 99.999%, ultra dry), lead(II) bromide (from Alfa Aesar, 99.999%), methylammonium iodide (from Dyesol Inc., 99.9%), methylammonium bromide (from Dyesol Inc., 99.9%), formadinium iodide (from Dyesol Inc.), cesium iodide (99.999%), chlorobenzene (sigma, anhydrous), 2,2',7,7'-tetrakis-(N,N-di-p-methoxyphenylamine)9,9'-spirobifluorene (spiro-OMeTAD).

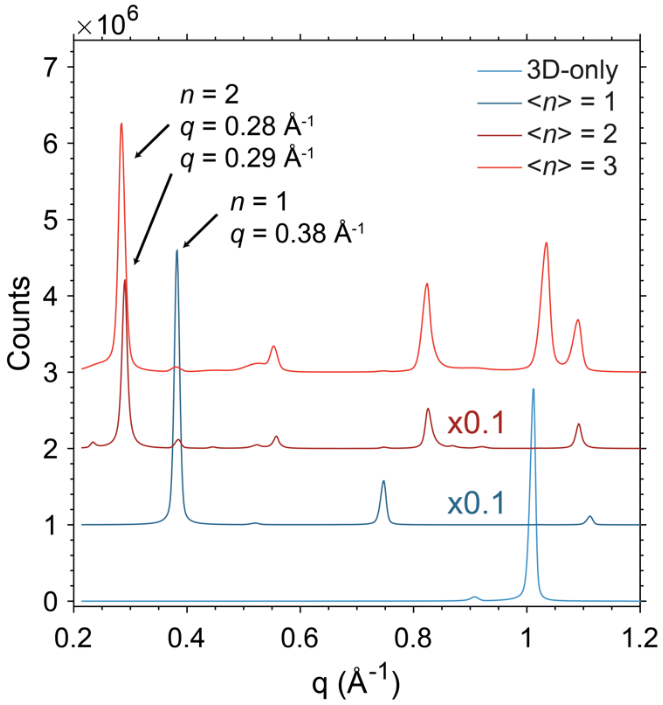

Supplementary Figure 1. XRD patterns for films of 3D-only and ⟨n⟩ = 1, 2, and 3 RDPs with composition $(VBABr)_2MA_{n-1}Pb_nI_{3n+1}$ ($MAPbI_3$ for 3D-only).

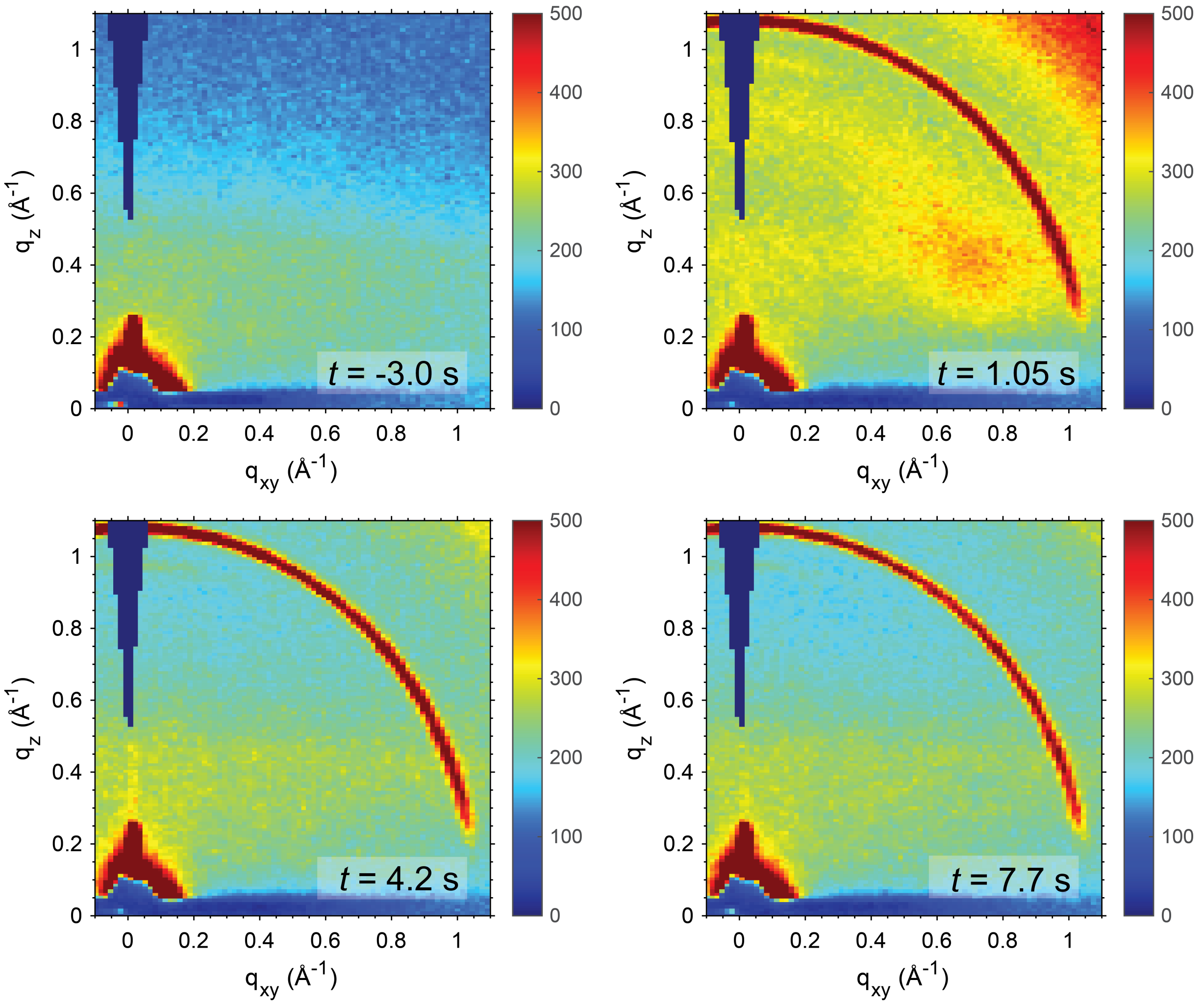

Supplementary Figure 2. *In situ* GIWAXS patterns for a film of $MAPbI_3$ exposed to pure IPA (no ligand). In the t = -3.0 s frame, the solvent is still soaking on the surface of the film, and so no diffraction is observed. The pattern after soaking still resembles untreated $MAPbI_3$, and no intermediate states at lower q values were observed.

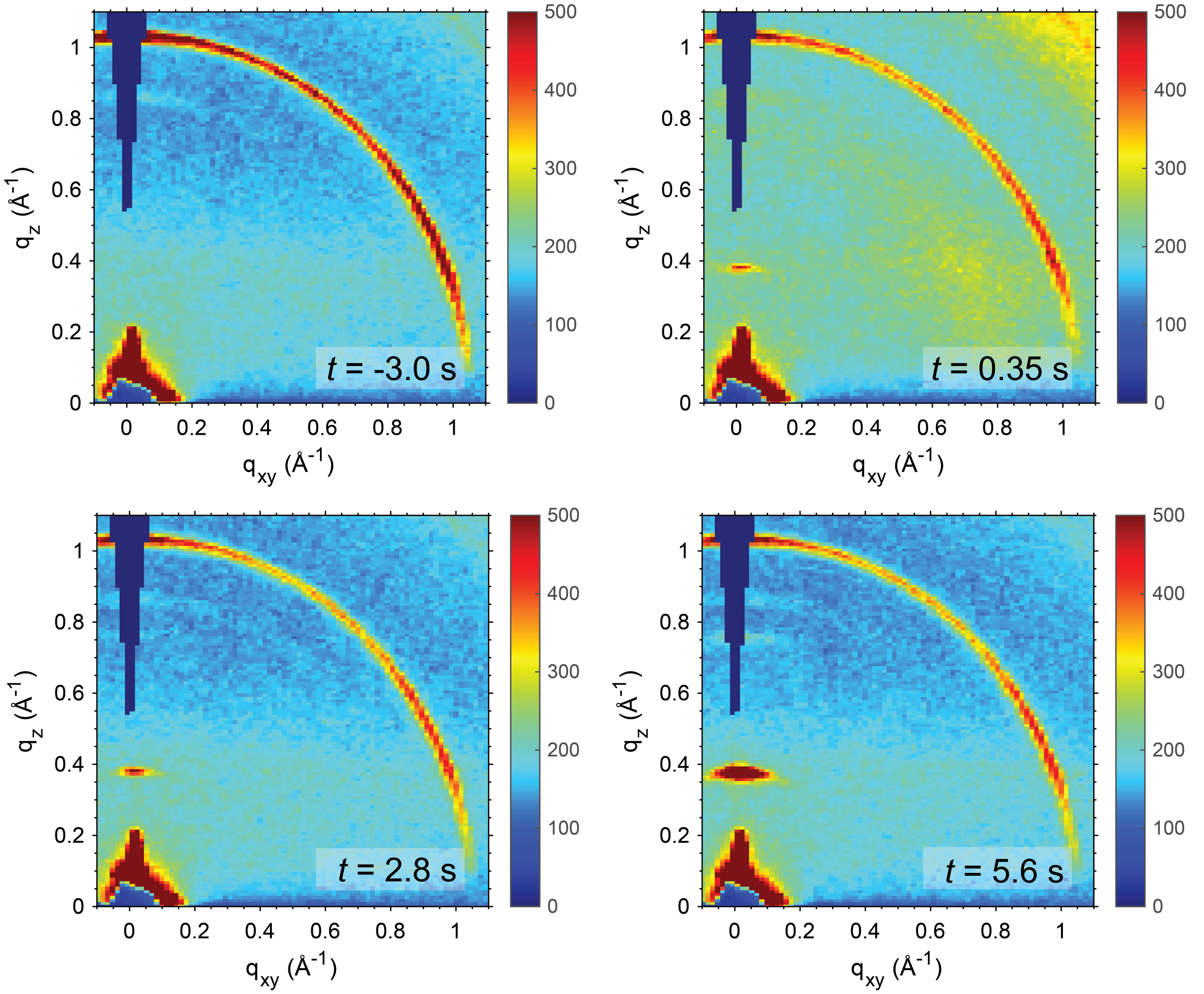

Supplementary Figure 3. *In situ* GIWAXS patterns of an $(MAPbBr_3)_{0.05}(FAPbI_3)_{0.95}$ film exposed to 5 mg/mL VBABr in IPA.

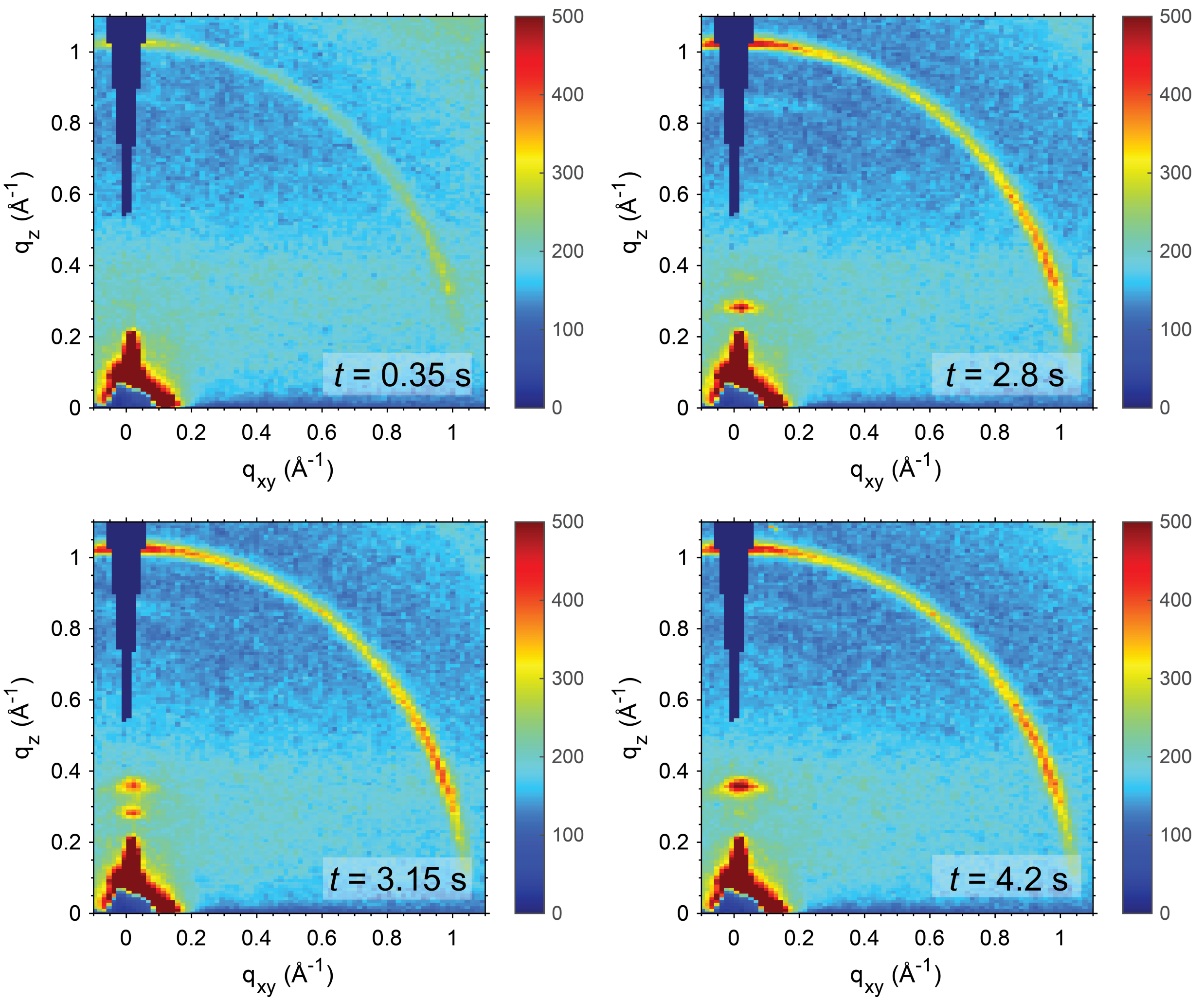

Supplementary Figure 4. *In situ* GIWAXS patterns of an $(MAPbBr_3)_{0.05}(FAPbI_3)_{0.95}$ film exposed to 5 mg/mL VBABr in 1:3 IPA:CB.

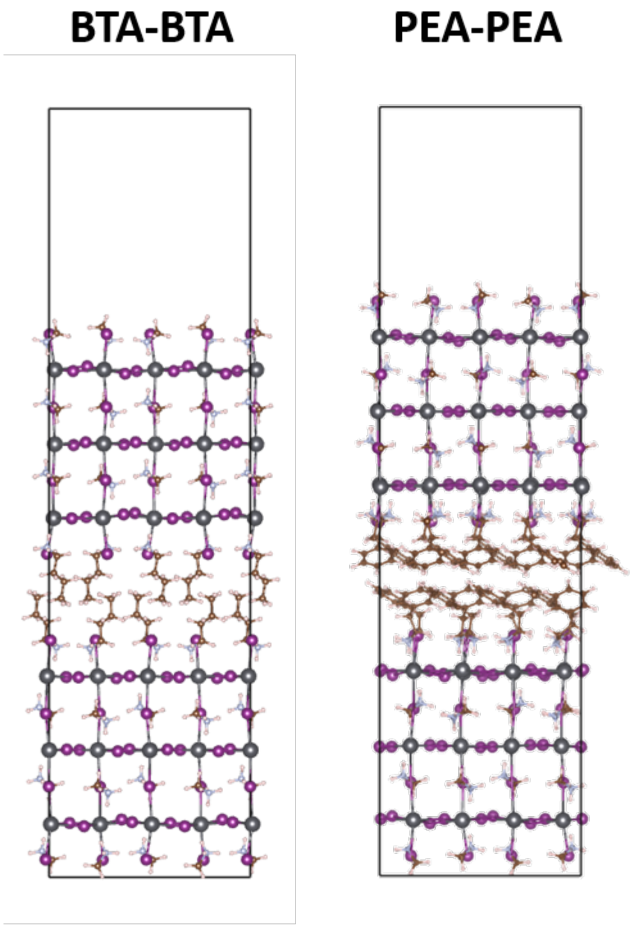

Supplementary Figure 5. DFT models used to calculate the binding energies of BTA-BTA and PEA-PEA interfaces.

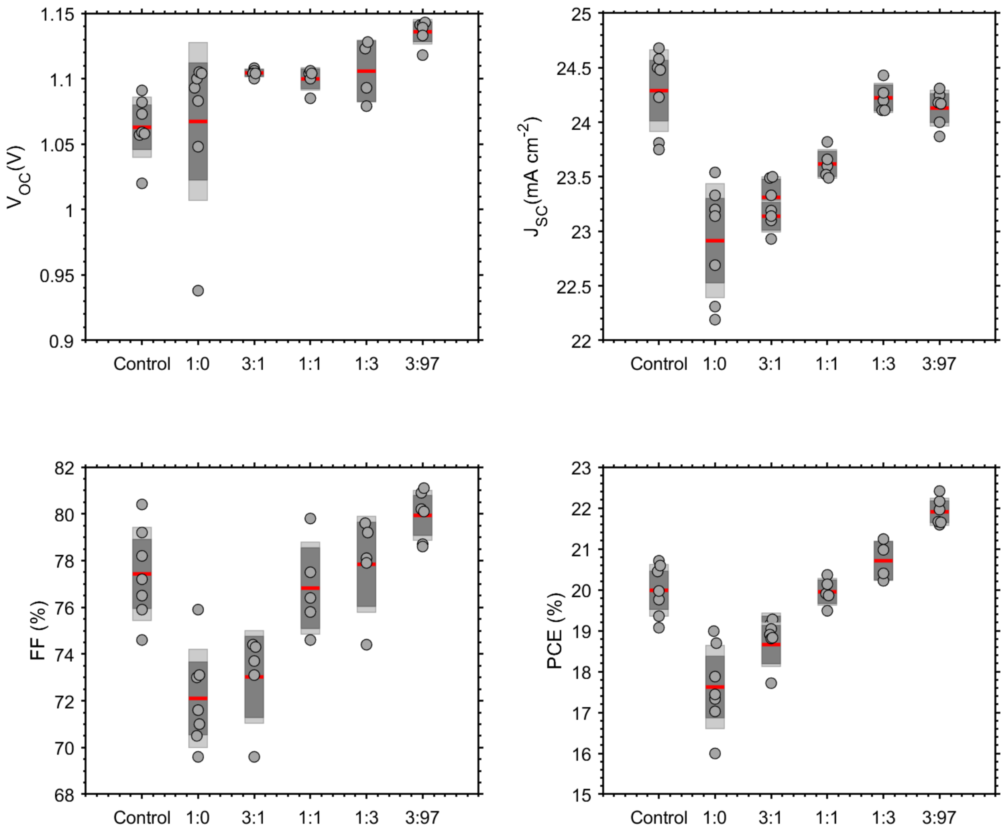

Supplementary Figure 6. Boxplots of V_{OC}, J_{SC}, FF, and PCE for different film treatment conditions. A tabulation of the different values and uncertainties can be found below (Supplementary Table 1).

	**Control**	**IPA:CB (1:0)**	**IPA:CB (3:1)**	**IPA:CB (1:1)**	**IPA:CB (1:3)**	**IPA:CF (3:97)**
**V_{OC} (V)**	1.06 ± 0.02	1.06 ± 0.07	1.10 ± 0.01	1.10 ± 0.01	1.10 ± 0.02	1.14 ± 0.01
**J_{SC} (mA cm^{-2})**	24.25 ± 0.40	22.87 ± 0.56	23.36 ± 0.17	23.62 ± 0.15	24.23 ± 0.15	24.16 ± 0.17
**PCE (%)**	19.93 ± 0.54	17.60 ± 1.46	18.73 ± 0.69	19.96 ± 0.37	21.06 ± 0.25	21.90 ± 0.28
**FF (%)**	77.67 ± 2.13	72.35 ± 2.17	72.65 ± 2.09	76.92 ± 2.25	78.70 ± 0.81	79.88 ± 1.16

Supplementary Table 1. Figures of merit averaged over multiple devices and associated standard deviations. Values were taken from reverse scanned *J-V* curves.

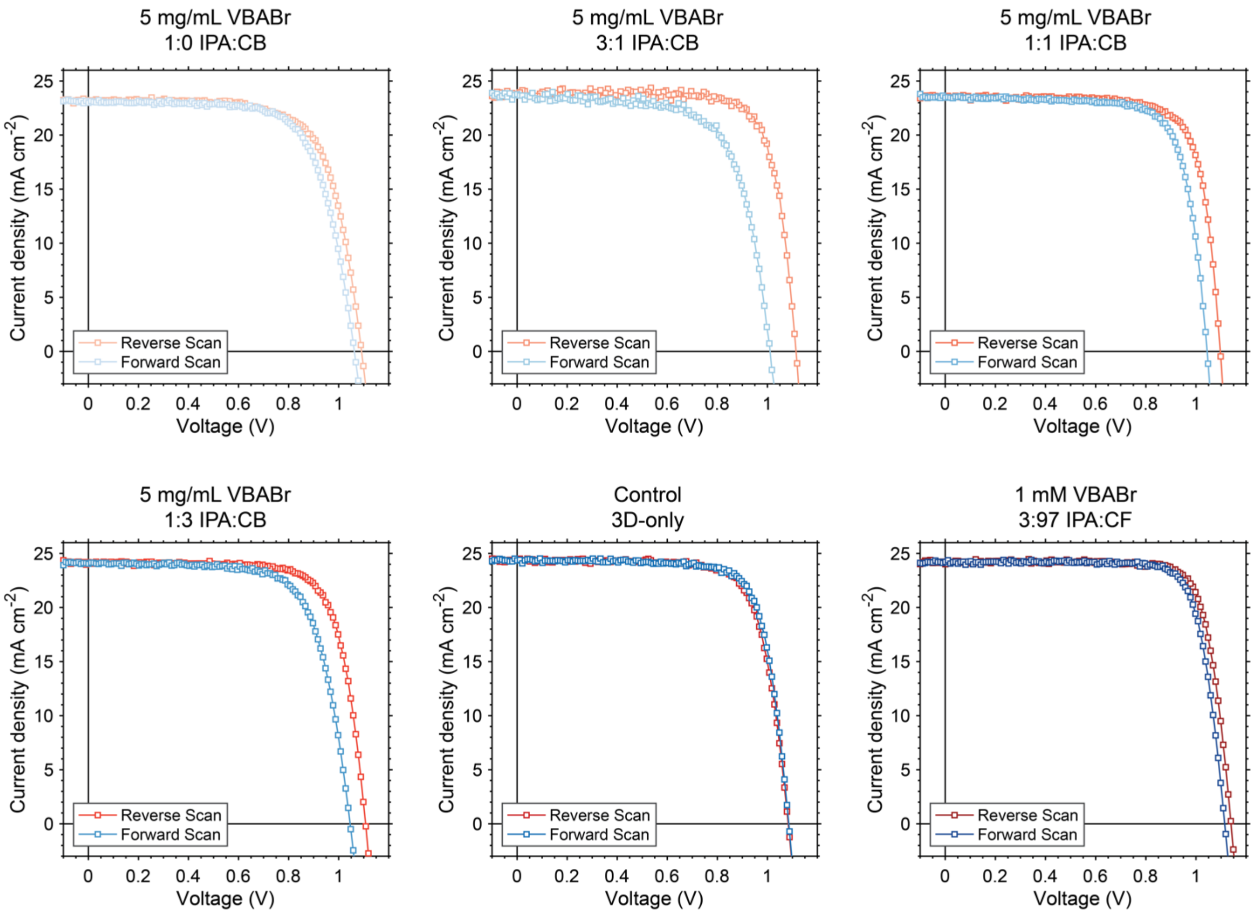

Supplementary Figure 7. Forward and reverse scan *J-V* curves for the same devices as in Figure 5 of the manuscript.

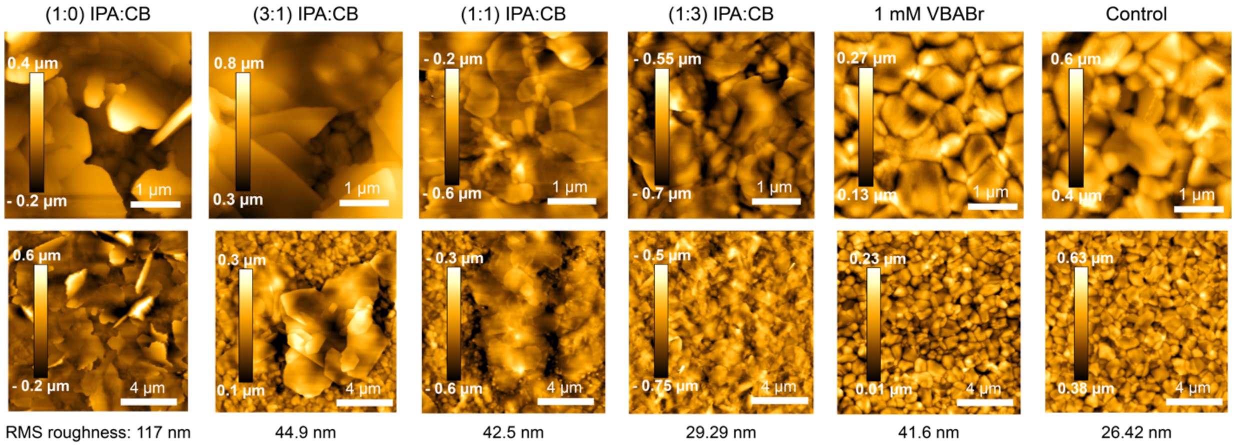

Supplementary Figure 8. AFM images for the 2D/3D and 3D control films scanned over different ranges.

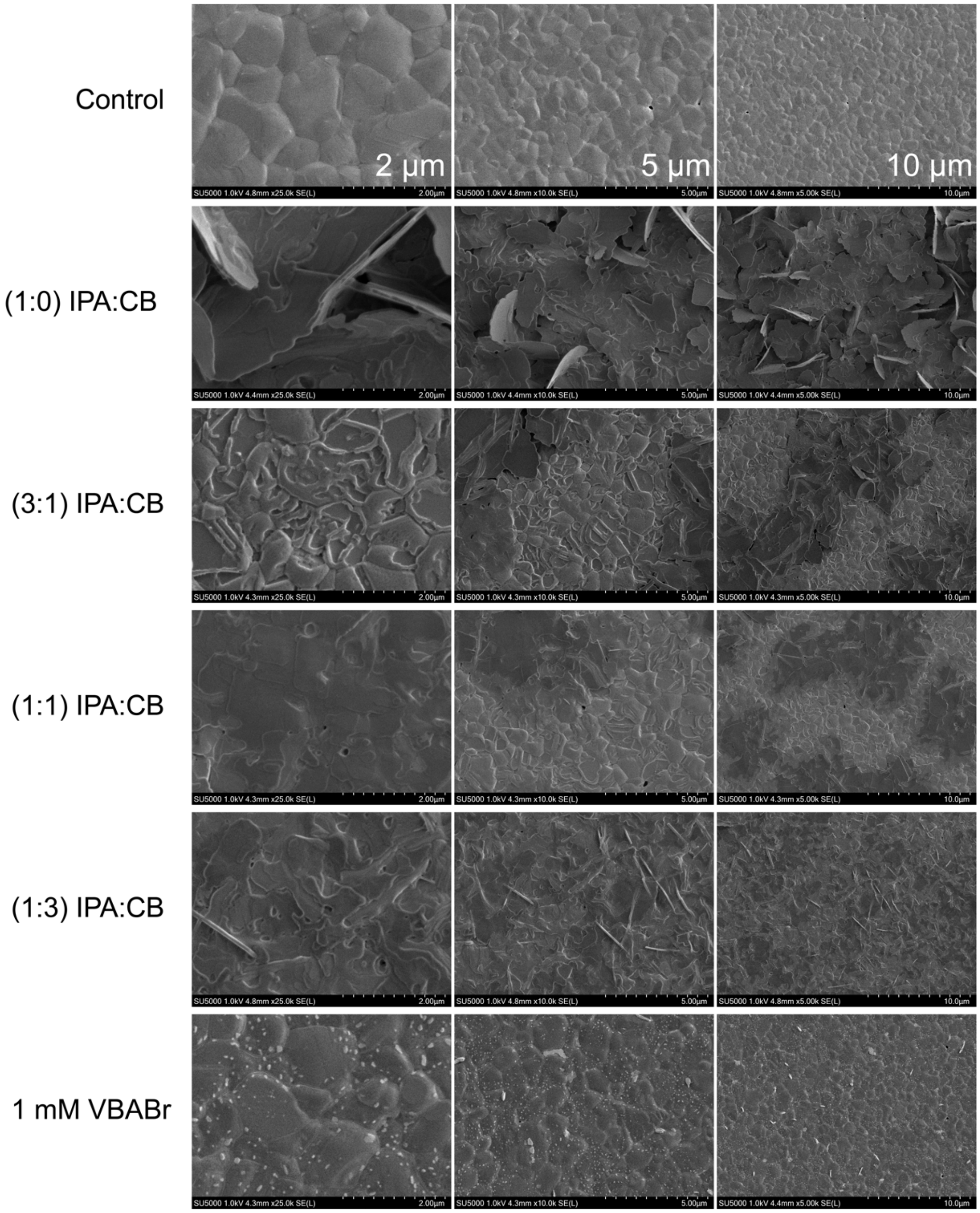

Supplementary Figure 9. SEM images for the 2D/3D and 3D control films at different magnifications.

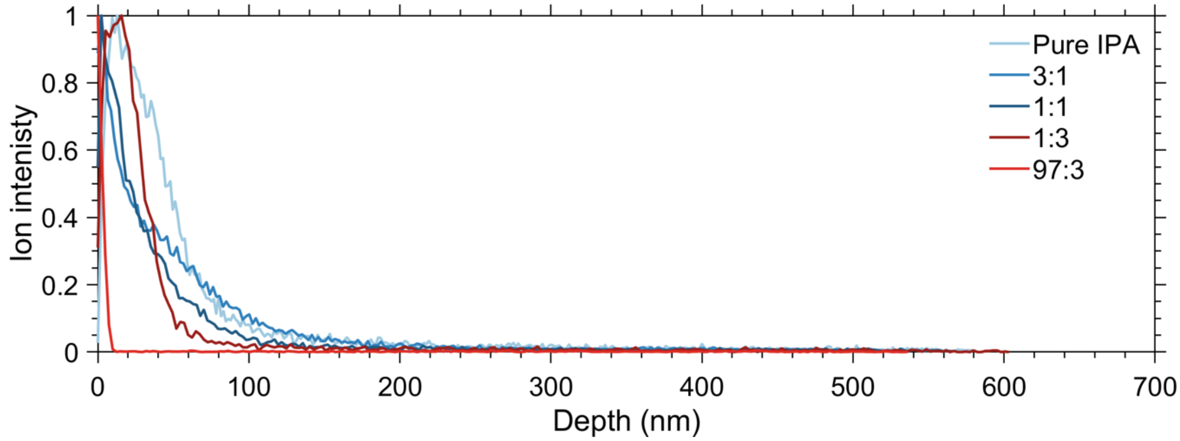

Supplementary Figure 10. ToF-SIMS depth profile of $C_9H_{12}N^+$ ions, corresponding to a fragment of the VBA ligand molecules.

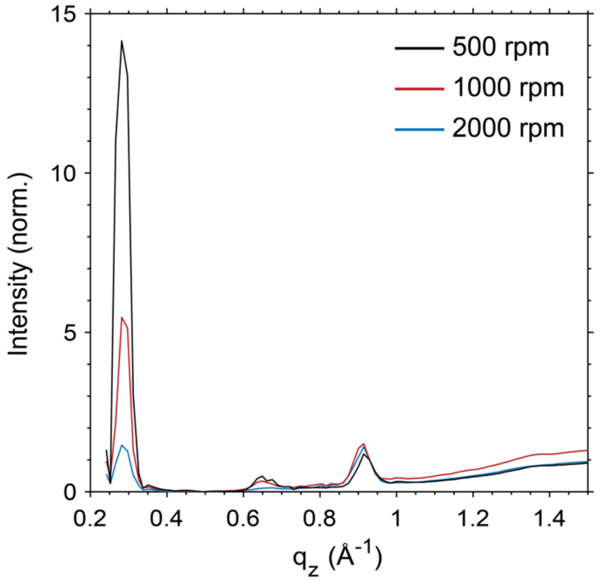

Supplementary Figure 11. Azimuthally integrated '*ex situ*' GIWAXS patterns (taken from the final frame of *in situ* experiments). Patterns are normalized to 1 at the 3D perovskite peak near $q_z \approx 0.9$ Å.

	A1	A2	T1	T2
**Control**	0.6586	-	618.3	-
	(0.6519, 0.6653)	-	(599.6, 637.1)	-
**Control + Spiro**	0.8976	-	28.35	-
	(0.8748, 0.9205)	-	(27.25, 29.46)	-
**IPA**	0.2954	0.674	312.5	860.4
	(0.2538, 0.337)	(0.6325, 0.7155)	(285.1, 340)	(828, 892.8)
**IPA + Spiro**	0.3157	0.6447	122.1	1770
	(0.3061, 0.3252)	(0.64, 0.6495)	(114.8, 129.4)	(1719, 1821)
**97:3 CF:IPA**	0.2153	0.9782	6.244	1846
	(0.1112, 0.3194)	(0.9733, 0.9831)	(3.329, 9.159)	(1819, 1872)
**97:3 CF:IPA + Spiro**	0.3205	0.4854	13.62	358.9
	(0.3072, 0.3338)	(0.4817, 0.489)	(12.67, 14.57)	(350.9, 366.9)
**3:1 CB:IPA**	0.1315	0.8368	111.5	1092
	(0.1247, 0.1382)	(0.8323, 0.8413)	(99.91, 123.2)	(1079, 1104)
**3:1 CB:IPA + Spiro**	0.2227	0.6854	117.9	798.6
	(0.2001, 0.2454)	(0.6676, 0.7031)	(98.38, 137.3)	(751.6, 845.6)
**1:3 CB:IPA**	0.1748	0.1748	78.23	616.9
	(0.1687, 0.1809)	(0.1687, 0.1809)	(72.78, 83.68)	(612.5, 621.2)
**1:3 CB:IPA + Spiro**	0.3285	0.6025	109.6	1796
	(0.3187, 0.3383)	(0.5979, 0.6071)	(103.2, 115.9)	(1743, 1849)
**1:1 CB:IPA**	0.08484	0.9151	154.7	1415
	(0.07728, 0.09241)	(0.9101, 0.9201)	(127.7, 181.7)	(1393, 1437)
**1:1 CB:IPA + Spiro**	0.3983	0.4837	130.4	1943
	(0.3869, 0.4097)	(0.4774, 0.49)	(123, 137.8)	(1847, 2039)

Supplementary Table 2. Fitted parameters for PL lifetime quenching experiments. Bracketed values below each fitted parameter are the 95% confidence intervals (as determined using MATLAB's default least squares fitting procedure).

Sample	Thickness (nm)
Pure IPA (5 mg/ml)	55
3:1 (IPA:CB) (5 mg/ml)	40
1:1 (IPA:CB) (5 mg/ml)	30
1:3 (IPA:CB) (5 mg/ml)	35
3:97 (IPA: CF) (1 mM)	4

Supplementary Table 3. Thickness of RDP layer in 2D/3D films estimated from 1/*e* of the TOF-SIMS profiles shown in Supplementary Figure 9.

Supplementary References

(1) Jung, E. H.; Jeon, N. J.; Park, E. Y.; Moon, C. S.; Shin, T. J.; Yang, T.-Y.; Noh, J. H.; Seo, J. Efficient, Stable and Scalable Perovskite Solar Cells Using Poly(3-Hexylthiophene). *Nature* **2019**, *567* (7749), 511. https://doi.org/10.1038/s41586-019-1036-3.

(2) Teale, S.; Proppe, A. H.; Jung, E. H.; Johnston, A.; Parmar, D. H.; Chen, B.; Hou, Y.; Kelley, S. O.; Sargent, E. H. Dimensional Mixing Increases the Efficiency of 2D/3D Perovskite Solar Cells. *J. Phys. Chem. Lett.* **2020**, *11* (13), 5115–5119. https://doi.org/10.1021/acs.jpclett.0c01444.

(3) Ilavsky, J. Nika: Software for Two-Dimensional Data Reduction. *J. Appl. Crystallogr.* **2012**, *45* (2), 324–328. https://doi.org/10.1107/S0021889812004037.

(4) Jiang, Z. GIXSGUI: A MATLAB Toolbox for Grazing-Incidence X-Ray Scattering Data Visualization and Reduction, and Indexing of Buried Three-Dimensional Periodic Nanostructured Films. *J. Appl. Crystallogr.* **2015**, *48* (3), 917–926. https://doi.org/10.1107/S1600576715004434.

(5) Giannozzi, P.; Baroni, S.; Bonini, N.; Calandra, M.; Car, R.; Cavazzoni, C.; Ceresoli, D.; Chiarotti, G. L.; Cococcioni, M.; Dabo, I.; Corso, A. D.; Gironcoli, S. de; Fabris, S.; Fratesi, G.; Gebauer, R.; Gerstmann, U.; Gougoussis, C.; Kokalj, A.; Lazzeri, M.; Martin-Samos, L.; Marzari, N.; Mauri, F.; Mazzarello, R.; Paolini, S.; Pasquarello, A.; Paulatto, L.; Sbraccia, C.; Scandolo, S.; Sclauzero, G.; Seitsonen, A. P.; Smogunov, A.; Umari, P.; Wentzcovitch, R. M. QUANTUM ESPRESSO: A Modular and Open-Source Software Project for Quantum Simulations of Materials. *J. Phys. Condens. Matter* **2009**, *21* (39), 395502. https://doi.org/10.1088/0953-8984/21/39/395502.

(6) Grimme, S.; Antony, J.; Ehrlich, S.; Krieg, H. A Consistent and Accurate Ab Initio Parametrization of Density Functional Dispersion Correction (DFT-D) for the 94 Elements H-Pu. *J. Chem. Phys.* **2010**, *132* (15), 154104. https://doi.org/10.1063/1.3382344.

(7) Perdew, J. P.; Burke, K.; Ernzerhof, M. Generalized Gradient Approximation Made Simple. *Phys. Rev. Lett.* **1996**, *77* (18), 3865–3868. https://doi.org/10.1103/PhysRevLett.77.3865.

(8) Vanderbilt, D. Soft Self-Consistent Pseudopotentials in a Generalized Eigenvalue Formalism. *Phys. Rev. B* **1990**, *41* (11), 7892–7895. https://doi.org/10.1103/PhysRevB.41.7892.
