# Supplementary material for: Multication perovskite 2D/3D interfaces form via progressive dimensional reduction
Source: Nat Commun. 2021 Jun 9;12:3472. doi: 10.1038/s41467-021-23616-9 (PMC8190276; doi:10.1038/s41467-021-23616-9)
Supplement: Supplementary file 3 — Solar Cells Reporting Summary [file 41467_2021_23616_MOESM3_ESM.pdf]

nature research

Corresponding author(s): Edward Sargent

Solar Cells Reporting Summary

Nature Research wishes to improve the reproducibility of the work that we publish. This form is intended for publication with all accepted papers reporting the characterization of photovoltaic devices and provides structure for consistency and transparency in reporting. Some list items might not apply to an individual manuscript, but all fields must be completed for clarity.

For further information on Nature Research policies, including our data availability policy, see Authors & Referees.

Experimental design

Please check: are the following details reported in the manuscript?

1. Dimensions

Item	Yes	No	Details
Area of the tested solar cells	☒	☐	Methods section, page 20
Method used to determine the device area	☒	☐	Methods section, page 20

2. Current-voltage characterization

Item	Yes	No	Details
Current density-voltage (J-V) plots in both forward and backward direction	☒	☐	Page S9 of the supporting information
Voltage scan conditions<br>*For instance: scan direction, speed, dwell times*	☒	☐	Methods section, page 20
Test environment<br>*For instance: characterization temperature, in air or in glove box*	☒	☐	Methods section, page 20
Protocol for preconditioning of the device before its characterization	☐	☒	There was no preconditioning of the device
Stability of the J-V characteristic<br>*Verified with time evolution of the maximum power point or with the photocurrent at maximum power point; see ref. 7 for details.*	☐	☒	We did not perform MPP tracking or stability studies for this work

3. Hysteresis or any other unusual behaviour

Item	Yes	No	Details
Description of the unusual behaviour observed during the characterization	☐	☐	*State where this information can be found in the text.*<br>*Explain why this information is not reported/not relevant.*
Related experimental data	☐	☐	*State where this information can be found in the text.*<br>*Explain why this information is not reported/not relevant.*

4. Efficiency

Item	Yes	No	Details
External quantum efficiency (EQE) or incident photons to current efficiency (IPCE)	☐	☒	EQE and IPCE were not relevant metrics to be compared in this work
A comparison between the integrated response under the standard reference spectrum and the response measure under the simulator	☐	☒	No EQEs were performed
For tandem solar cells, the bias illumination and bias voltage used for each subcell	☐	☒	No tandems were reported in this work

5. Calibration

Item	Yes	No	Details
Light source and reference cell or sensor used for the characterization	☒	☐	Methods section, page 20
Confirmation that the reference cell was calibrated and certified	☒	☐	Methods section, page 20

Calculation of spectral mismatch between the reference cell and the devices under test

☒ Yes — Methods section, page 20
☐ No

6. Mask/aperture

Size of the mask/aperture used during testing

☒ Yes — Methods section, page 20
☐ No

Variation of the measured short-circuit current density with the mask/aperture area

☐ Yes
☒ No — We have a fixed aperture size that we do not vary

7. Performance certification

Identity of the independent certification laboratory that confirmed the photovoltaic performance

☐ Yes — *State where this information can be found in the text.*
☐ No — *Explain why this information is not reported/not relevant.*

A copy of any certificate(s)
Provide in Supplementary Information

☐ Yes — *State where this information can be found in the text.*
☐ No — *Explain why this information is not reported/not relevant.*

8. Statistics

Number of solar cells tested

☒ Yes — Page S14 of supporting information
☐ No

Statistical analysis of the device performance

☒ Yes — Page S14 of supporting information
☐ No

9. Long-term stability analysis

Type of analysis, bias conditions and environmental conditions
For instance: illumination type, temperature, atmosphere humidity, encapsulation method, preconditioning temperature

☐ Yes
☒ No — We did not perform long-term stability tests in this work
